# Analysis of Article 6 (tax and price measures to reduce the demand for tobacco products) of the WHO Framework Convention on Tobacco Control

**DOI:** 10.1136/tobaccocontrol-2018-054462

**Published:** 2018-07-25

**Authors:** Corne van Walbeek, Samantha Filby

**Affiliations:** 1 School of Economics, University of Cape Town, Rondebosch, South Africa; 2 Southern Africa Labour and Development Research Unit, University of Cape Town, Rondebosch, South Africa

**Keywords:** economics, taxation, global health

## Abstract

**Objective:**

To analyse the extent to which parties to the WHO Framework Convention on Tobacco Control (FCTC) have implemented Article 6 since the convention’s entry into force.

**Methods:**

Compliance was measured using nine indicators, derived from the 2016 version of the FCTC’s reporting instrument’s core questionnaire, and the WHO’s MPOWER cigarette affordability measure. Data were collected from WHO country profiles, and the 12 country mission reports by the Impact Assessment Expert Group.

**Results:**

The number of parties reporting any type of excise tax increased from 87% (134/154) in 2008 to 92% (160/174) in 2016. Specific excise tax systems were implemented by 36% (63/174) of FCTC ratifying countries in 2016, up from 32% (49/154) in 2008. The proportion of parties with mixed tax structures has increased from 25% (39/154) in 2008 to 32% (56/174) in 2016. The proportion of parties that levy the tax as a fully ad valorem tax has decreased from 29% (45/154) in 2008 to 24% (42/174) in 2016. Cigarettes have become less affordable in 46% (78/168), more affordable in 13% (21/168) and unchanged in terms of affordability in 41% (69/168) of parties between 2008 and 2016. The number of parties that earmark tobacco tax revenues for public health increased from 13 in 2008 to 30 in 2016. Many finance ministries are hesitant to increase the excise tax, mainly due to illicit trade concerns.

**Conclusion:**

While there has been some improvement in tobacco tax policy over time, parties should adopt stronger tax measures, despite industry opposition and threats about illicit trade. Parties should implement FCTC Article 5.3 and ratify the Protocol to Eliminate Illicit Trade in Tobacco Products.

## Introduction

The World Health Organisation’s Framework Convention on Tobacco Control (WHO FCTC, hereafter FCTC) is the world’s first public health treaty, and became effective on 27 February 2005.^1^ It was developed with the objective of protecting current and future generations from the harmful effects of tobacco use, and exposure to tobacco smoke.^2^ The FCTC recommends that ratifying countries adopt policy measures to reduce both the demand for (Articles 6–14) and supply of (Articles 15–17) tobacco products.^2^ These recommendations are based on scientific evidence, best practice and the experience of countries that have effectively implemented tobacco control policies in ways that have improved the health of their populations. To date, 181 parties—representing 90% of the world’s population—have ratified the treaty.^3^

The year 2015 marked the milestone of 10 years since the FCTC’s entry into force.^4^ To commemorate the occasion, the Conference of the Parties (COP) mandated the formation of an Expert Group to determine the extent to which the FCTC had enabled countries to implement effective tobacco control policies in its first decade.^5^

Article 6 of the FCTC commits parties to using tax and price policies to reduce tobacco use.

To assist parties in their implementation of Article 6, guidelines for its implementation were adopted by the COP at its sixth session in October 2014.^6^ The Article 6 guidelines recommend, among other things, that parties implement simple tobacco taxation systems by adopting specific or mixed excise systems (as opposed to purely ad valorem systems), that tax rates be adjusted regularly to account for inflation and income growth, that tax rates be applied uniformly to all products, and that a proportion of tobacco tax revenues collected be earmarked to finance tobacco control.^6^

Tax and price policies are widely recognised as the most effective means of influencing the demand for—and thus the consumption of—tobacco products.^7–9^ As a consequence, implementation of FCTC Article 6 is an essential element of a country’s tobacco control strategy and public health improvement efforts. In spite of this, the evidence review found that global progress in the implementation of Article 6 has advanced slowly in comparison to other substantive articles of the FCTC.^4^ The purpose of this paper is to analyse the impact of the FCTC on the adoption of national tobacco taxation policies, in accordance with Article 6, and to identify barriers that prevent better tax structures and higher tax levels.

## Data and methods 

We track countries’ compliance with nine indicators drawn from the core questionnaire of the 2016 FCTC Reporting Instrument administered by WHO FCTC Secretariat.^10^ The instrument measures the extent to which Article 6 has been implemented between 2007 (the year of the first FCTC reporting cycle) and 2016 (the year of the most recent FCTC reporting cycle). In addition to the indicators derived from the FCTC reporting instrument, we also use the WHO’s MPOWER measure for ‘cigarette affordability’ in our analysis.^11^ This is because questions on cigarette affordability were only introduced to the FCTC reporting instrument in 2016, and studies have shown that affordability is at least equal to—or more relevant than—the tax burden (tax as a percentage of retail price) to evaluate the effectiveness of tobacco tax policies.^12 13^

Table 1 lists the 10 indicators that we used to assess the impact of the FCTC on the adoption of FCTC-compliant tax and price policies since the treaty’s entry into force. These indicators were chosen because countries’ compliance with each indicator has been tracked consistently—both by the WHO and the FCTC Convention Secretariat—since the FCTC’s entry into force, and these indicators reflect the major provisions with which countries are expected to comply. The chosen indicators allow for an analysis of changes in tobacco taxation systems, changes in the tobacco tax burden and cigarette affordability, as well as the earmarking of tobacco taxes for financing of tobacco control and other public health initiatives.

**Table 1:** List of indicators used

Topic	Indicators
Tax structure	Only specific tax levied
	Only ad valorem tax levied
	Combination of specific and ad valorem taxes levied
Trends in taxation and prices	Proportion of the retail price consisting of taxes
	Trends in taxation
	Trends in retail prices
	WHO MPOWER affordability measure
Other indicators used	Tobacco tax earmarking
	Progress made in implementing Article 6
	Additional information concerning price and tax measures

To measure compliance with each of these indicators, we drew on data compiled by the WHO’s biennial Global Report on the Tobacco Epidemic.^11 14–17^ These data provide measures of countries’ compliance with each of the above indicators for all even-numbered years between (and including) 2008 and 2016. Given our focus on the FCTC and its implementation, our initial intention was to use information generated by the parties in their implementation reports submitted to the FCTC Convention Secretariat. These reports are available on the Secretariat’s online implementation database.^18^ However, these data presented two problems which prompted us to seek an alternative source.

First, the number of observations in the Secretariat’s database is contingent on parties submitting their implementation reports and answering all questions on the relevant indicators in those reports. Because the number of observations for each indicator is contingent on parties’ reporting, we risked overestimating compliance if countries do not report on an issue because they are non-compliant. The extent of the potential bias is made evident when one considers that, in 2012, only 98 of the 174 parties to the FCTC (56%) provided a report which answered the question that measured compliance with the recommendation in Article 6 that countries implement an excise tax policy to reduce the demand for tobacco.

The data underpinning WHO’s Global Report on the Tobacco Epidemic, on the other hand, are obtained independently by WHO and do not rely on countries’ commitment to fill out the reporting instrument. This mitigates the potential for biases in our measurement of compliance due to parties’ selective reporting.

Second, we found discrepancies in parties’ implementation reports for some indicators, which also prompted us to seek an alternative source. Parties’ implementation reports are usually completed by an official in the ministry of health, who may have limited understanding of the excise tax regimen. For example, of the six African (AFRO) countries that reported the tax burden on cigarettes in both 2014 and 2016, three countries (Ghana, Madagascar and Mali) reported unrealistic declines (from 88% to 30%, 73.5% to 20% and 79.7% to 35%, respectively), which raised questions about the accuracy of the reported tax burdens, not only for these three countries, but generally.

We also noticed that, in their first implementation report, several countries wrongly reported that they did not have an excise tax, whereas in fact they did. Using such data would bias our conclusions regarding the association between FCTC ratification and the adoption of tobacco excise taxation regimens. It would wrongly create the impression that the adoption of the FCTC was associated with an increase in the number of countries that have adopted an excise tax system. Because WHO data do not rely on self-reporting, the potential for such discrepancy is reduced.

Besides addressing the two concerns outlined above, WHO data offered the added benefit of including trends in cigarette affordability over time, an indicator only introduced to the FCTC reporting instrument in 2016. Because WHO’s sample is global, as opposed to being confined to only FCTC ratifying countries, we tailored WHO’s compliance data to reflect only those countries who had ratified the FCTC in each reporting year. Data on the status of FCTC ratification were obtained from the United Nations Treaty Section.^19^ Table 2 summarises the maximum potential sample on which we could report for each indicator.

**Table 2:** Number of countries that have ratified the FCTC, 2008–2016

Region	2008	2010	2012	2014	2016
Africa (AFRO)	36	40	40	43	43
Americas (AMRO)	26	28	29	30	30
Eastern Mediterranean (EMRO)	17	19	19	19	19
Europe (EURO)	44	46	49	50	50
South-East Asia (SEARO)	10	10	10	10	10
Western Pacific (WPRO)	27	27	27	27	27
Total	160	170	174	179	179

Source, constructed based on the FCTC ratification status listed in the United Nations Treaty Section.

All countries who had ratified the FCTC by the relevant year are included in the sample.

FCTC, Framework Convention on Tobacco Control.

In addition to the trend analysis of the adoption of FCTC-compliant price and tax policies over time, we also include anecdotal evidence from the 12 country missions undertaken by the seven-member WHO FCTC Impact Assessment Expert Group. These missions occurred between November 2015 and May 2016. While Article 6 of the FCTC addresses the taxation of all tobacco products, we limit our analysis to trends in the taxation and price of cigarettes, on account of the paucity of data on non-cigarette tobacco products.

## Results

### Tax structure

Overall, the total number of parties that reported levying some form of excise tax on tobacco products increased from 134 of 154 parties (87%) in 2008 to 160 of 174 parties (92%) in 2016 (table 3).

**Table 3:** Percentage of parties that levy an excise tax on cigarettes, by WHO region

Region	2008	2010	2012	2014	2016
AFRO	94% (34/36)	95% (37/39)	92% (36/39)	95% (40/42)	98% (39/40)
AMRO	92% (24/26)	96% (27/28)	93% (27/29)	93% (28/30)	97% (29/30)
EMRO	47% (8/17)	47% (9/19)	47% (9/19)	44% (8/18)	68% (13/19)
EURO	100% (43/43)	100% (46/46)	100% (49/49)	100% (50/50)	100% (50/50)
SEARO	86% (6/7)	86% (6/7)	88% (7/8)	78% (7/9)	78% (7/9)
WPRO	76% (19/25)	75% (18/24)	81% (21/26)	88% (22/25)	85% (22/26)
Total	87% (134/154)	88% (144/164)	88% (149/170)	89% (155/174)	92% (160/174)

Source, estimates constructed from data contained in the individual tobacco control ‘country profiles’ of FCTC ratifying countries published by WHO in 2009, 2011, 2013, 2015 and 2017.

All percentages rounded to the nearest whole number; EURO region does not include the European Union.

FCTC, Framework Convention on Tobacco Control.

Eight of the 20 parties that did not levy any form of excise tax in 2008 had introduced excise taxes subsequently. Of these eight parties, three introduced a purely ad valorem system (Mauritania, Iraq and Grenada) and five implemented a purely specific excise taxation system (Bahrain, Iran, Saudi Arabia, Kiribati and Palau). As of 2016, only 12 countries (six from the Eastern Mediterranean (EMRO), one from South-East Asia (SEARO), one from the Americas (AMRO), one from Africa (AFRO) and three from the Western Pacific (WPRO)) do not report any excise taxes, but levy only value added taxes and import duties. None of these 12 countries produce cigarettes locally, which means that the import duty becomes the de facto excise tax. Table 4 offers a breakdown of the types of excise tax levied by each WHO region over time.

**Table 4:** Type of excise tax regimen, by WHO region

WHO Region	2008	2010	2012	2014	2016
AFRO	(n=36)	(n=39)	(n=39)	(n=42)	(n=40)
Specific only	33%	31%	26%	29%	28%
Ad valorem only	58%	62%	64%	60%	58%
Combination of specific and ad valorem	3%	3%	3%	7%	13%
No excise tax	6%	5%	8%	5%	3%
AMRO	(n=26)	(n=28)	(n=29)	(n=30)	(n=30)
Specific only	38%	46%	41%	43%	47%
Ad valorem only	54%	39%	28%	27%	27%
Combination of specific and ad valorem	0%	11%	24%	23%	23%
No excise tax	8%	4%	7%	7%	3%
EMRO	(n=17)	(n=19)	(n=19)	(n=18)	(n=19)
Specific only	12%	5%	5%	17%	32%
Ad valorem only	24%	21%	21%	17%	26%
Combination of specific and ad valorem	12%	21%	21%	11%	11%
No excise tax	53%	53%	53%	56%	32%
EURO	(n=43)	(n=46)	(n=49)	(n=50)	(n=50)
Specific only	23%	20%	18%	22%	22%
Ad valorem only	2%	4%	6%	4%	4%
Combination of specific and ad valorem	74%	76%	76%	74%	74%
No excise tax	0%	0%	0%	0%	0%
SEARO	(n=7)	(n=7)	(n=8)	(n=9)	(n=9)
Specific only	29%	14%	25%	22%	44%
Ad valorem only	29%	29%	25%	22%	11%
Combination of specific and ad valorem	29%	43%	38%	33%	22%
No excise tax	14%	14%	13%	22%	22%
WPRO	(n=25)	(n=24)	(n=26)	(n=25)	(n=26)
Specific only	56%	50%	58%	64%	65%
Ad valorem only	12%	13%	12%	12%	12%
Combination of specific and ad valorem	8%	13%	12%	12%	8%
No excise tax	24%	25%	19%	12%	15%
Total	(n=154)	(n=164)	(n=170)	(n=174)	(n=174)
Specific only	32%	30%	29%	33%	36%
Ad valorem only	29%	28%	26%	25%	24%
Combination of specific and ad valorem	25%	30%	32%	32%	32%
No excise tax	13%	12%	12%	11%	8%

Source, Estimates constructed from data contained in the individual tobacco control ‘country profiles’ of FCTC ratifying countries published by WHO in 2009, 2011, 2013, 2015 and 2017.

All percentages rounded to the nearest whole number; EURO region does not include the European Union. The sum of the percentages may not add to 100 due to rounding.

FCTC, Framework Convention on Tobacco Control.

Globally, mixed tax systems (ie, a combination of specific and ad valorem taxes) have become more widely used over time, increasing from 25% in 2008 to 32% in 2016. Specific excise tax systems remain the most popular, having increased from 32% in 2008 to 36% in 2008. The number of parties that levy the tax as a fully ad valorem tax has decreased from 29% in 2008 to 24% in 2016.

The predominant taxation regimen varies by region. Parties from the EURO region favour a combination of specific and ad valorem taxes. Countries in the European Union are bound by the EU directives which mandate a mixed tax system. Parties in the Western Pacific region have a preference for specific tax only, whereas African parties have a strong preference for ad valorem tax systems.

While some parties (eg, Chile and Costa Rica between 2012 and 2014) have made progress in simplifying their tax structures by relying more on specific taxes,^20^ others have not. Tiered taxes, which impose differentiated tax rates based on some characteristics of the product, were still used in 31 of the 159 parties (19%) for which these data were available in 2016. Notable parties that have changed their tax structure in the past decade to reflect the FCTC’s recommendations include Pakistan^21^ and the Philippines.^22^ In 2013, both parties replaced their complex, multitiered tax systems with simplified specific tax systems that are easier to implement, improve health outcomes, maximise revenue collected and reduce the likelihood of tobacco industry manipulation to circumvent tax increases.^21 22^

## Trends in taxation and prices

Table 5 shows the mean and median share of excise taxes in the price of a pack of the most-sold brand of cigarettes, by WHO region, between 2008 and 2016. We also include the interquartile range (IQR) as a measure of spread. Globally, there has been an increase in the share of the excise taxes in the price of a pack of the most-sold brand of cigarettes over the period of interest. The mean share of excise taxes has gradually increased from 33% of the retail price in 2008, to 36% of the retail price in 2016. The mean (and median) excise tax share increased in the WPRO and EMRO regions, but it remained largely constant in the AFRO and AMRO regions. With a median excise tax share of 58% in 2016, the EURO region comes closest to the 70% target set in the Article 6 guidelines. With a median excise tax burden of 17% and 20%, respectively, in 2016, EMRO and AFRO are farthest from the Article 6 target.

**Table 5:** Excise tax share in the price of a pack* of the most-sold brand of cigarettes between 2008 and 2016, by WHO region

Region	2008	2010	2012	2014	2016
AFRO	(n=36)	(n=39)	(n=39)	(n=42)	(n=40)
Mean	23%	24%	21%	23%	24%
Median	16%	17%	16%	18%	20%
IQR	23%	25%	24%	20%	19%
AMRO	(n=26)	(n=28)	(n=29)	(n=30)	(n=30)
Mean	32%	33%	32%	32%	34%
Median	30%	34%	30%	31%	33%
IQR	26%	24%	35%	31%	27%
EMRO	(n=17)	(n=19)	(n=19)	(n=18)	(n=19)
Mean	19%	22%	23%	21%	26%
Median	0%	0%	0%	0%	17%
IQR	33%	39%	52%	43%	44%
EURO	(n=43)	(n=46)	(n=49)	(n=50)	(n=50)
Mean	49%	52%	51%	51%	51%
Median	57%	59%	58%	57%	58%
IQR	19%	11%	12%	16%	13%
SEARO	(n=7)	(n=7)	(n=8)	(n=9)	(n=9)
Mean	34%	36%	38%	34%	31%
Median	29%	29%	40%	30%	26%
IQR	36%	37%	35%	43%	37%
WPRO	(n=25)	(n=24)	(n=26)	(n=25)	(n=26)
Mean	28%	29%	32%	36%	36%
Median	30%	28%	29%	42%	37%
IQR	47%	50%	42%	34%	35%
Global	(n=154)	(n=164)	(n=170)	(n=174)	(n=174)
Mean	33%	34%	34%	35%	36%
Median	32%	34%	33%	34%	36%
IQR	44%	45%	45%	43%	41%

Source, estimates constructed from data contained in the individual tobacco control ‘country profiles’ of FCTC ratifying countries published by WHO in 2009, 2011, 2013, 2015 and 2017.

*20 pieces; all percentages rounded to the nearest whole number; EURO region does not include the European Union.

FCTC, Framework Convention on Tobacco Control.

Retail prices of cigarettes have shown an increase over the same period (table 6). Globally, the median retail price on the most-sold brand increased from 3.10 international dollars in 2008, to 4.94 international dollars in 2016. As at 2016, the highest prices are observed in the EURO region, followed by WPRO and SEARO. The lowest prices are observed in the AFRO region, where the median price is 3.42 international dollars.

**Table 6:** Price of a pack* of the most-sold brand of cigarettes in international dollars (adjusted for purchasing power parity) between 2008 and 2016, by WHO region

Region	2008	2010	2012	2014	2016
AFRO	(n=36)	(n=38)	(n=38)	(n=41)	(n=39)
Mean	2.97	3.32	3.06	3.49	3.82
Median	2.48	2.72	2.25	2.57	3.42
IQR	1.76	1.88	1.51	1.60	2.25
AMRO	(n=26)	(n=28)	(n=29)	(n=30)	(n=30)
Mean	3.49	3.51	4.54	5.04	5.92
Median	3.26	2.77	4.32	4.26	4.76
IQR	1.61	1.71	1.83	2.08	3.04
EMRO	(n=17)	(n=17)	(n=18)	(n=18)	(n=18)
Mean	2.46	2.74	3.11	3.58	4.53
Median	2.53	2.75	3.28	3.87	3.83
IQR	1.17	1.53	1.58	2.28	2.90
EURO	(n*=*43)	(n=46)	(n=49)	(n=50)	(n=50)
Mean	4.08	4.53	5.18	5.87	6.71
Median	4.00	4.72	5.42	6.11	6.66
IQR	2.10	2.56	3.39	3.33	3.09
SEARO	(n=8)	(n=8)	(n=8)	(n=8)	(n=8)
Mean	4.10	4.10	4.68	5.24	7.00
Median	3.77	3.37	3.94	4.31	5.39
IQR	2.34	2.41	2.49	3.38	4.30
WPRO	(n=22)	(n=21)	(n=24)	(n=23)	(n=24)
Mean	4.15	4.29	4.95	5.21	6.61
Median	4.03	3.84	4.18	4.25	5.54
IQR	3.29	3.21	4.12	3.98	7.15
Global	(n=152)	(n=161)	(n=166)	(n=170)	(n=169)
Mean	3.55	3.87	4.30	4.80	5.67
Median	3.10	3.39	3.70	4.18	4.94
IQR	2.32	2.58	3.43	3.71	4.24

Source, estimates constructed from data contained in the individual tobacco control ‘country profiles’ of FCTC ratifying countries published by WHO in 2009, 2011, 2013, 2015 and 2017.

*20 pieces; all values rounded to two decimal places; EURO region does not include the European Union.

FCTC, Framework Convention on Tobacco Control.

**Figure 1:**
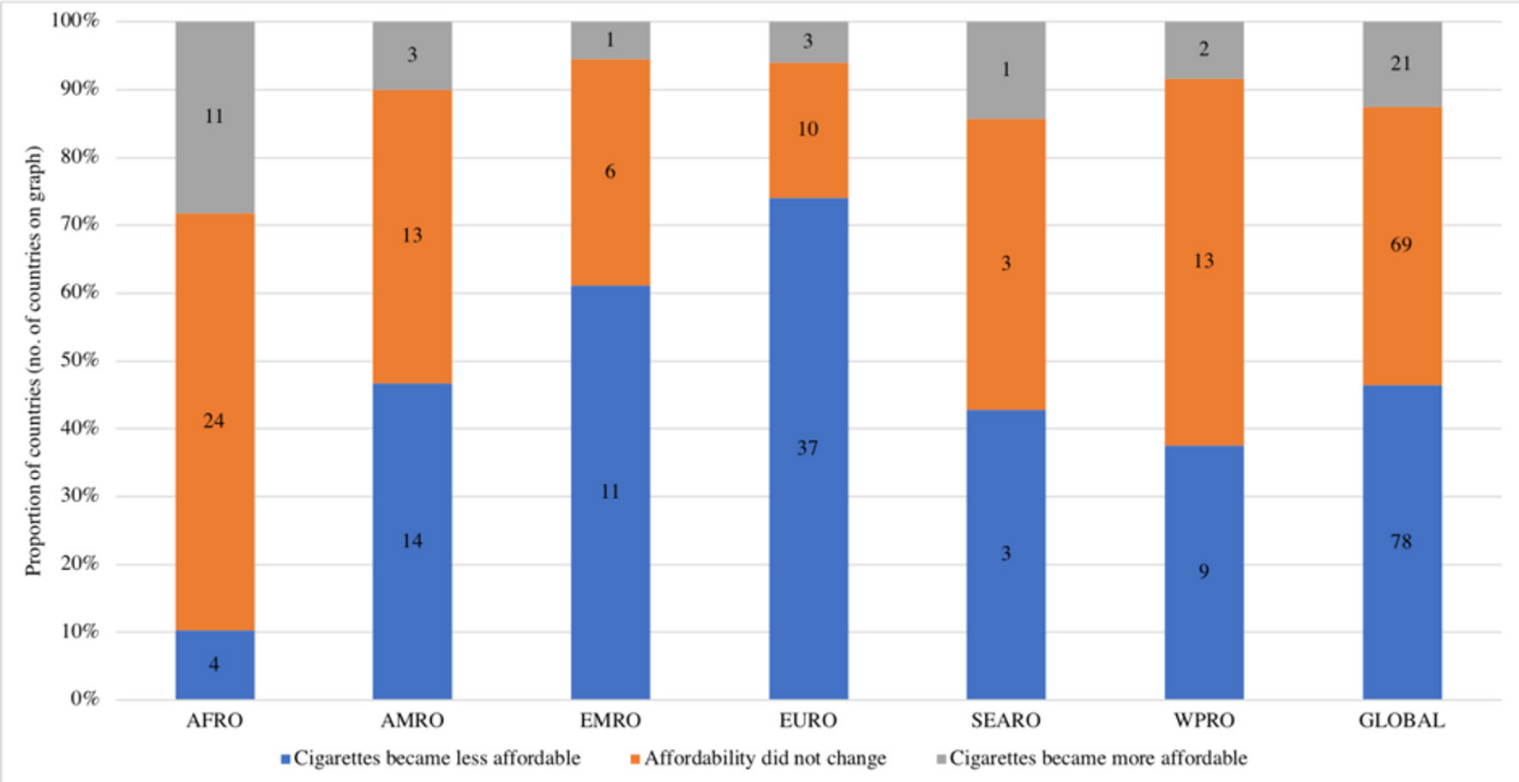
Cigarette affordability trends, 2008–2016 (n=168). Source: estimates constructed from information contained in the individual country profiles compiled by WHO 2017. This figure only shows information for those countries that had ratified the FCTC by November 2016. FCTC, Framework Convention on Tobacco Control.

The relationship between excise taxes and the retail price (ie, the tax burden) and trends in the retail price are two dimensions of the effectiveness of tax policy. Changes in tobacco taxes are only effective as a tobacco control tool if they raise the retail price of cigarettes so that cigarettes become less affordable over time.^11^ Tobacco products become less affordable if the price of cigarettes increases by a greater percentage than the sum of the inflation rate and the growth in per capita income, over time.^11^ Figure 1 shows changes in cigarette affordability between 2008 and 2016 for the 168 parties that had comparable data over this period, where cigarette affordability is measured as the percentage of per capita GDP required to purchase 100 packs of 20 cigarettes.

Of the 168 parties, cigarettes became less affordable in 46% (78/168), remained constant in 41% (69/168), but became more affordable in 13% (21/168) of parties. Eleven of the 21 parties in which cigarettes became more affordable over the 2008–2016 period are from the African region.

Although cigarette affordability has decreased, or remained the same, in most parties since 2008 (figure 1), many parties do not have a policy of consistently reducing the affordability of cigarettes over time. We find that, of the 72 parties for which cigarettes had become less affordable between 2012 and 2014, cigarettes became more affordable in 33 of those parties (46%) between 2014 and 2016. This illustrates the need for automatic adjustments in taxes to account for changes in inflation and income growth.

As at 2016, only 15 parties automatically adjust their specific excise tax to account for inflation. For example, in 2012 the Philippines introduced its ‘Sin Tax’ reform, which substantially simplified and increased the excise tax between 2013 and 2017, and provided for an automatic annual 4% increase in cigarette taxes after 2018.^23^ In December 2013, Australia implemented the first of four preannounced annual 12.5% tobacco excise tax increases, in addition to biannual inflation adjustments.^24^ In 2016, the Australian government announced a further four rounds of 12.5% annual increases in tobacco excise, which began in July 2017.^25^

### Earmarking tobacco taxes

The purpose of tobacco tax earmarking is to dedicate a portion of taxes or revenues from tobacco taxation to health and/or tobacco control. Earmarking partially corrects the negative externality of tobacco use.^26^ In 2008, 13 of the 160 parties (8%) earmarked tobacco taxes for health purposes. As of 2016, 30 parties (16% of ratifying countries) dedicate (at least a portion of) tobacco tax revenue for health purposes. Only 10 of these 30 parties (6% of ratifying countries) dedicate a portion of this revenue to tobacco control specifically.

### Anecdotes from impact assessment country visits

Twelve countries were visited by the WHO FCTC Impact Assessment Expert Group between November 2015 and May 2016. While most discussions were with officials from the ministries of health and other health-focused groups, the Expert Group also had discussions with officials from the ministries of finance and customs, the revenue authority and groups with an economic focus.

Asked which FCTC article is regarded the single most important and influential article, most respondents from the health ministry identified Article 8 (smoke-free policies) as the most important. This may stem from the ‘visibility’ of smoke-free policies. Smoke-free laws are immediately visible as a smoke-free environment, especially when these laws are enforced effectively. According to these officials, smoke-free policies act as a foundation on which other tobacco control interventions, including increases in the excise tax, can be built.

While there was agreement that increasing the excise tax is an important tobacco control instrument, health ministry officials often had a very cursory understanding of the more technical aspects of the tax system, for example, the structure of the excise tax. Excise tax was often perceived as a finance issue, not a health issue. In many countries, the relationship between the health ministry and the finance ministry (and related government institutions) is quite strained.

Finance ministries indicated that it was often difficult to raise the excise tax. Sometimes the legislative process to change the excise tax is onerous. Often the threat of illicit trade was raised as a reason why the excise tax should not be increased. The influence of the tobacco industry in dissuading ministries of finance from increasing the excise tax was very obvious in some countries.

These barriers notwithstanding, the FCTC has contributed to progress in the implementation of price and tax measures to reduce the demand for tobacco. For example, the Expert Group found that following FCTC ratification, the UK adopted two sets of regulations with the intention of supporting health objectives. These are the duty escalator for cigarettes and hand-rolled tobacco products, adopted in 2010, and the rebalancing of cigarette duties in 2011 to reduce price differentials and combat downtrading. These regulations have given the UK some of the highest cigarette taxes and prices in the EU, and the world.

## Discussion

Despite the fact that increasing the excise tax is generally regarded as the single most effective tobacco control intervention, parties to the FCTC have implemented Article 6 at a substantially lower rate than other FCTC articles.^4^ Whereas Article 8 (smoke-free policies) and Article 11 (banning of tobacco advertising, promotion and sponsorship) have average implementation rates of 88% and 76%, respectively, the average implementation rate for Article 6 stands at only at 65%.^i^ This is cause for concern.

The Expert Group found that the effectiveness of increasing the excise tax is often not appreciated by government officials. Whereas non-tax tobacco control measures are typically once-off interventions (eg, smoke-free policies or advertising bans, once they are implemented and enforced, cannot be implemented again), this is not true of raising the excise tax. There is no logical upper limit to the excise tax. A few parties, like Australia and the Philippines, have adopted policies to preannounce automatic and substantial increases in the excise tax for a number of years. The prospect of continuously increasing tobacco prices acts as a strong incentive for smokers to quit smoking, continuing smokers to smoke less and non-smokers to not start smoking.

The structure of tobacco taxes implemented in a country has important implications for both public health and public finance.^27^ The most appropriate tax structure is one which reduces tobacco consumption, minimises the opportunities for downtrading, and increases government revenue. Generally, specific excise taxes are simpler to administer and offer a more predictable revenue stream than ad valorem taxes.^27^ Increasing specific excise taxes also result in fairly uniform price increases across all the tobacco product categories, whereas ad valorem excise taxes can create large price differentials between (and within) tobacco product categories.^27^ The only drawback of specific taxes is that they can be eroded by inflation, which necessitates regular increases in the nominal excise tax.

Taking cognisance of a large and growing literature on the importance of the structure of the excise tax, the guidelines to Article 6 encourage parties to adopt simple tax structures. Parties are advised to increase the specific component of the excise tax and decrease the ad valorem component. The specific component should be adjusted regularly to take account of inflation and income growth. It is encouraging that countries are moving away from purely ad valorem taxes to either mixed tax systems or specific taxes. Africa is in the most vulnerable position of all WHO regions in the matter of excise taxation structure as it has the largest proportion of countries with ad valorem rates.

While tobacco taxes are widely supported, earmarking is a contentious topic. In some countries earmarking of tobacco taxes is anathema, while in other countries earmarking a proportion of the revenue for public health or poverty alleviation projects makes raising the excise tax more politically palatable. The fact that there has been just over a doubling between 2010 and 2016 of the number of countries that earmark tobacco revenues means that earmarking is becoming more mainstream.

The study has limitations. In the absence of a counterfactual or a sizeable control group, one is unable to ascribe causality to the observed progress made in the implementation of Article 6 to the FCTC’s efforts. The most that can be inferred is a strong association. Additionally, while Article 6 is concerned with price and tax measures used to reduce demand for all tobacco products, our analysis was confined to cigarettes. Information on the tax regimens of tobacco products other than cigarettes is often sparse and inadequate. The lack of this information prevents an assessment of the impact of the FCTC on price and taxation policies in relation to such products. Data collection needs to be intensified for other tobacco products.

Given that this study’s focus was on how the FCTC supported countries in their implementation of better tax structures and policies, the most appropriate data, a priori, would have been the parties’ submissions to the FCTC Secretariat through the reporting instrument. However, the quality of the self-reported tax and price data in these reports were of such variable quality that we had no choice, but to use data from the WHO’s Global Report on the Tobacco Epidemic. While the Secretariat offers support to parties in fulfilling their reporting obligations through the provision of step-by-step instructions on completion of the reporting instrument, there is a need for more direct support to be offered to parties, specifically in respect of Article 6.

Despite these limitations, our results suggest that there has been some progress in tax policy and tax levels, over time, but that there is much scope for further improvement. Anecdotal evidence from the in-country visits indicates that tobacco industry interference is a major impediment to more aggressive excise taxation within countries. Article 5.3 of the FCTC obligates parties to protect the formulation and implementation of public health policies for tobacco control from the tobacco industry.^2^ Therefore, to counter industry interference, countries should implement Article 5.3 of the FCTC as a means to support progress in relation to Article 6 of the FCTC.

Moreover, since the central tenet of the tobacco industry’s claims against higher excise taxes is that higher taxes lead to an increase in illicit trade, ratification of the Protocol to Eliminate Illicit Trade in Tobacco Products, the first protocol to the FCTC, adopted in November 2012, is crucial in order to provide ministries of finance with the scope and confidence to increase the excise tax burden more aggressively, to achieve both public health and fiscal gains.

**What this paper adds**

**What is already known on this subject**

- In 2015/2016, WHO Framework Convention on Tobacco Control (FCTC) Impact Assessment Expert Group performed a study to determine to what extent the FCTC had enabled countries to implement effective tobacco control policies.
- There is consensus that increase in the price of cigarettes is the single most effective way of reducing tobacco consumption and smoking prevalence.
- The FCTC recommends that countries adopt simple tax structures, rather than complex tax structures. Specifically, a uniform specific tax that is adjusted regularly to take account of inflation and income increases, is regarded as the gold standard.

**What important gaps in knowledge exist on this topic**

- To what extent, if at all, has the FCTC been successful in supporting parties to implement effective tobacco tax policies in accordance to the principles of Article 6 and the associated guidelines?
- What are some of the main impediments that prevent parties from implementing higher taxes?

**What this paper adds**

- Although some parties have been successful in implementing effective tobacco tax policies in its first 10 years, progress overall has been slow.

## Footnotes

i Average implementation rates for each article are calculated by the FCTC Convention Secretariat as the percentage of the reporting parties that provided an affirmative answer in respect of the indicators used to measure implementation of a particular FCTC article.^10^

## References

[1] Roemer R, Taylor A, Lariviere J Origins of the WHO Framework Convention on Tobacco Control. Am J Public Health 2005;95:936–8. 10.2105/AJPH.2003.025908 15914812 PMC1449287

[2] World Health Organization. The WHO Framework Convention on Tobacco Control. Geneva, Switzerland: World Health Organization, 2003 http://apps.who.int/iris/bitstream/10665/42811/1/9241591013.pdf (26 Mar 2018).

[3] World Health Organization Framework Convention on Tobacco Control Convention Secretariat. Parties to the WHO Framework Convention on Tobacco Control. 2018 http://www.who.int/fctc/cop/en/.

[4] Chung-Hall J, Craig L, Fong GT, et al Impact of the WHO Framework Convention on Tobacco Control on the Implementation and Effectiveness of Tobacco Control Measures: A Global Evidence Review. Waterloo, Ontario, Canada: ITC Project, University of Waterloo, 2016.

[5] World Health Organization Framework Convention on Tobacco Control Conference of the Parties. Impact Assessment of the WHO FCTC: Report by the Expert Group. *FCTC/COP/7/6* . 2016 http://www.who.int/fctc/cop/cop7/FCTC_COP_7_6_EN.pdf (30 Mar 2018).

[6] World Health Organization Convention on Tobacco Control Conference of the Parties. Guidelines for the implementation of Article 6 of the WHO FCTC (Decision FCTC/COP6(5)). Geneva, Switzerland: World Health Organization Press, 2014 http://www.who.int/fctc/guidelines/adopted/Guidelines_article_6.pdf (2018, March 17).

[7] Jha P, Chaloupka FJ Curbing the Epidemic: Governments and the Economics of Tobacco Control. Washington, DC: World Bank, 1999.

[8] International Agency for Research on Cancer. 2011. Effectiveness of Tax and Price Policies for Tobacco Control. IARC Handbooks of Cancer Prevention, Tobacco Control. 14 Lyon, France: International Agency for Research on Cancer https://www.iarc.fr/en/publications/pdfs-online/prev/handbook14/handbook14.pdf (30 Aug 2017).

[9] United States National Cancer Institute and World Health Organization. The Economics of Tobacco and Tobacco Control. National Cancer Institute Tobacco Control Monograph 21. NIH Publication No. 16-CA-8029A. Bethesda, MD: U.S. Department of Health and Human Services, National Institutes of Health, National Cancer Institute; and Geneva, CH: World Health Organization., 2017.

[10] World Health Organization Framework Convention on Tobacco Control Convention Secretariat. Global Progress Report on implementation of the WHO Framework Convention on Tobacco Control. Geneva: WHO Press, 2016 http://www.who.int/fctc/reporting/2016globalprogressreport.pdf (30 Mar 2018).

[11] World Health Organisation. WHO Report on the Global Tobacco Epidemic. 2017 http://www.who.int/tobacco/global_report/en/ (19 Mar 2018).

[12] Blecher EH, van Walbeek CP An international analysis of cigarette affordability. Tob Control 2004;13:339–46. 10.1136/tc.2003.006726 15564616 PMC1747952

[13] Blecher E Targeting the affordability of cigarettes: a new benchmark for taxation policy in low-income and-middle-income countries. Tob Control 2010;19:325–30. 10.1136/tc.2009.030155 20530141

[14] World Health Organisation. WHO Report on the Global Tobacco Epidemic. 2009 http://www.who.int/tobacco/global_report/en/ (19 Mar 2018).

[15] World Health Organisation. WHO Report on the Global Tobacco Epidemic. 2011 http://www.who.int/tobacco/global_report/en/ (19 Mar 2018).

[16] World Health Organisation. WHO Report on the Global Tobacco Epidemic. 2013 http://www.who.int/tobacco/global_report/en/ (19 Mar 2018).

[17] World Health Organisation. WHO Report on the Global Tobacco Epidemic. 2015 http://www.who.int/tobacco/global_report/en/ (19 Mar 2018).

[18] World Health Organization Framework Convention on Tobacco Control Convention Secretariat. WHO FCTC Implementation Database. 2018 http://www.who.int/fctc/reporting/implement_database/en/ (30 May 2018).

[19] United Nations Treaty Collection. 2018. *World Health Organization Framework Convention on Tobacco Control* . Available 2018 https://treaties.un.org/pages/.

[20] World Health Organization Framework Convention on Tobacco Control Convention Secretariat. Global Progress Report on implementation of the WHO Framework Convention on Tobacco Control. Geneva: WHO Press, 2014 http://www.who.int/fctc/reporting/2014globalprogressreport.pdf?ua=1 (30 Mar 2018).

[21] Baloch A, et al The economics of tobacco and tobacco taxation in Pakistan. Paris: The Union, 2013.

[22] Chaloupka FJ, et al The economics of tobacco and tobacco taxation in the Philippines. Paris: The Union, 2012.

[23] Southeast Asia Initiative on Tobacco Tax of the Southeast Asian Tobacco Control Alliance. ASEAN tobacco tax report card: regional comparisons and trends. 5th ed: SEATCA, 2014 https://seatca.org/dmdocuments/ASEANTaxReportCard%20Sep14%20(1).pdf (11 Mar 2018).

[24] Australian National Department of Health. Taxation: the history of tobacco excise arrangements in Australia since 1901: Government of Australia, 2014 http://www.health.gov.au/internet/main/publishing.nsf/content/tobacco-tax (2 Apr 2018).

[25] Thomas M Tobacco Excise Increase: Budget Review 2016-2017 Index. 2017 https://www.aph.gov.au/About_Parliament/Parliamentary_Departments/Parliamentary_Library/pubs/rp/BudgetReview201617/Tobacco (7 Apr 2018).

[26] Marquez PV Expanding the global tax base. Taxing to promote public goods: tobacco taxes. Panel session held as part of Winning the Tax Wars: Global Solutions for Developing Countries. Summary report. Washington: World Bank Group Conference, 2016 http://documents.worldbank.org/curated/en/820951485943150390/pdf/112347-WP-web-copy-PUBLIC.pdf (24 May 2016 11Mar 2018).

[27] World Health Organization. Tobacco tax levels and structure: a theoretical and empirical overview: Types of taxes levied on tobacco products. Geneva Switzerland: WHO Technical Manual on Tax Administration WHO Press, 2010 http://apps.who.int/bookorders/anglais/detart1.jsp?codlan=1&codcol=15&codcch=786&content=1 (08 May 2018).

